# Supplementary material for: Musculoskeletal Disorder Symptoms among Workers at an Informal Electronic-Waste Recycling Site in Agbogbloshie, Ghana
Source: Int J Environ Res Public Health. 2021 Feb 19;18(4):2055. doi: 10.3390/ijerph18042055 (PMC7923259; doi:10.3390/ijerph18042055)
Supplement: Supplementary file 1 [file ijerph-18-02055-s001.zip › ijerph-1046873-suppl.docx]

**Table S1.** Total counts for musculoskeletal discomfort frequency, severity, and work interference ratings strat-ified by body part and primary job category. Total N for severity and work interference ratings are based on participants with a non-zero discomfort frequency rating.

| Body Part | Primary Job Category | Discomfort Frequency Rating Count | | | | | |  | Discomfort Severity Rating Count | | | |  | Discomfort Interference with Work Count | | | |
| --- | --- | --- | --- | --- | --- | --- | --- | --- | --- | --- | --- | --- | --- | --- | --- | --- | --- |
|  |  | **N** | **Never** | **1-2 times last week** | **3-4 times  last week** | **Once every day** | **Several times every day** |  | **N*** | **Slightly uncomfortable** | **Moderately uncomfortable** | **Very uncomfortable** |  | **N*** | **Not at all** | **Slightly interfered** | **Substantially interfered** |
| **Neck** | Collectors | 73 | 55 | 2 | 6 | 9 | 1 |  | 18 | 9 | 5 | 4 |  | 18 | 4 | 5 | 9 |
|  | Dismanters | 82 | 59 | 7 | 7 | 8 | 1 |  | 23 | 6 | 6 | 11 |  | 23 | 9 | 9 | 5 |
|  | Burners | 21 | 16 | 2 | 3 | 0 | 0 |  | 5 | 2 | 2 | 1 |  | 5 | 2 | 3 | 0 |
|  | Reference group | 41 | 32 | 4 | 4 | 1 | 0 |  | 10 | 5 | 4 | 1 |  | 8 | 5 | 3 | 0 |
|  | Combined | 217 | 162 | 15 | 20 | 18 | 2 |  | 56 | 22 | 17 | 17 |  | 54 | 20 | 20 | 14 |
| **Shoulder, right** | Collectors | 73 | 47 | 3 | 6 | 16 | 1 |  | 28 | 11 | 6 | 11 |  | 26 | 8 | 9 | 9 |
|  | Dismanters | 82 | 48 | 14 | 9 | 9 | 2 |  | 34 | 19 | 8 | 7 |  | 33 | 16 | 8 | 9 |
|  | Burners | 21 | 15 | 3 | 2 | 1 | 0 |  | 6 | 2 | 2 | 2 |  | 6 | 0 | 6 | 0 |
|  | Reference group | 41 | 28 | 8 | 1 | 4 | 0 |  | 14 | 7 | 4 | 3 |  | 11 | 2 | 9 | 0 |
|  | Combined | 217 | 138 | 28 | 18 | 30 | 3 |  | 82 | 39 | 20 | 23 |  | 76 | 26 | 32 | 18 |
| **Upper back** | Collectors | 73 | 64 | 1 | 4 | 3 | 1 |  | 9 | 3 | 0 | 6 |  | 8 | 2 | 4 | 2 |
|  | Dismanters | 82 | 67 | 4 | 6 | 3 | 2 |  | 16 | 6 | 4 | 6 |  | 17 | 8 | 5 | 4 |
|  | Burners | 21 | 19 | 1 | 1 | 0 | 0 |  | 3 | 1 | 1 | 1 |  | 3 | 3 | 0 | 0 |
|  | Reference group | 41 | 35 | 1 | 3 | 1 | 1 |  | 7 | 1 | 4 | 2 |  | 6 | 1 | 5 | 0 |
|  | Combined | 217 | 185 | 7 | 14 | 7 | 4 |  | 35 | 11 | 9 | 15 |  | 34 | 14 | 14 | 6 |
| **Upper arm, right** | Collectors | 73 | 51 | 3 | 8 | 10 | 1 |  | 23 | 10 | 5 | 8 |  | 23 | 5 | 9 | 9 |
|  | Dismanters | 82 | 55 | 12 | 7 | 7 | 1 |  | 28 | 13 | 11 | 4 |  | 27 | 13 | 8 | 6 |
|  | Burners | 21 | 20 | 1 | 0 | 0 | 0 |  | 0 | 0 | 0 | 0 |  | 0 | 0 | 0 | 0 |
|  | Reference group | 41 | 40 | 0 | 1 | 0 | 0 |  | 1 | 1 | 0 | 0 |  | 1 | 1 | 0 | 0 |
|  | Combined | 217 | 166 | 16 | 16 | 17 | 2 |  | 52 | 24 | 16 | 12 |  | 51 | 19 | 17 | 15 |
| **Lower back** | Collectors | 73 | 24 | 9 | 10 | 28 | 2 |  | 51 | 13 | 9 | 29 |  | 50 | 20 | 15 | 15 |
|  | Dismanters | 82 | 26 | 16 | 19 | 14 | 7 |  | 55 | 13 | 18 | 24 |  | 54 | 26 | 19 | 9 |
|  | Burners | 21 | 10 | 6 | 2 | 3 | 0 |  | 11 | 4 | 1 | 6 |  | 11 | 2 | 6 | 3 |
|  | Reference group | 41 | 20 | 7 | 4 | 6 | 4 |  | 23 | 8 | 3 | 12 |  | 17 | 8 | 7 | 2 |
|  | Combined | 217 | 80 | 38 | 35 | 51 | 13 |  | 140 | 38 | 31 | 71 |  | 132 | 56 | 47 | 29 |
| **Forearm, right** | Collectors | 73 | 70 | 1 | 0 | 1 | 1 |  | 3 | 2 | 1 | 0 |  | 3 | 1 | 2 | 0 |
|  | Dismanters | 82 | 79 | 2 | 1 | 0 | 0 |  | 4 | 1 | 2 | 1 |  | 4 | 0 | 4 | 0 |
|  | Burners | 21 | 19 | 1 | 1 | 0 | 0 |  | 2 | 0 | 2 | 0 |  | 2 | 1 | 1 | 0 |
|  | Reference group | 41 | 41 | 0 | 0 | 0 | 0 |  | 0 | 0 | 0 | 0 |  | 0 | 0 | 0 | 0 |
|  | Combined | 217 | 209 | 4 | 2 | 1 | 1 |  | 9 | 3 | 5 | 1 |  | 9 | 2 | 7 | 0 |
| **Wrist & hand, right** | Collectors | 73 | 68 | 2 | 0 | 2 | 1 |  | 6 | 6 | 0 | 0 |  | 4 | 3 | 0 | 1 |
|  | Dismanters | 82 | 70 | 6 | 1 | 4 | 1 |  | 12 | 8 | 2 | 2 |  | 12 | 7 | 3 | 2 |
|  | Burners | 21 | 18 | 1 | 1 | 1 | 0 |  | 3 | 1 | 0 | 2 |  | 2 | 0 | 1 | 1 |
|  | Reference group | 41 | 38 | 1 | 1 | 1 | 0 |  | 3 | 1 | 1 | 1 |  | 3 | 2 | 1 | 0 |
|  | Combined | 217 | 194 | 10 | 3 | 8 | 2 |  | 24 | 16 | 3 | 5 |  | 21 | 12 | 5 | 4 |
| **Wrist & hand, left** | Collectors | 73 | 67 | 2 | 0 | 2 | 2 |  | 6 | 6 | 0 | 0 |  | 4 | 2 | 0 | 2 |
|  | Dismanters | 82 | 70 | 5 | 1 | 4 | 2 |  | 12 | 8 | 2 | 2 |  | 12 | 7 | 3 | 2 |
|  | Burners | 21 | 18 | 1 | 1 | 1 | 0 |  | 3 | 1 | 0 | 2 |  | 2 | 0 | 1 | 1 |
|  | Reference group | 41 | 39 | 0 | 1 | 1 | 0 |  | 2 | 0 | 1 | 1 |  | 2 | 1 | 1 | 0 |
|  | Combined | 217 | 194 | 8 | 3 | 8 | 4 |  | 23 | 15 | 3 | 5 |  | 20 | 10 | 5 | 5 |
| **Hip & buttocks** | Collectors | 73 | 71 | 0 | 1 | 0 | 1 |  | 2 | 2 | 0 | 0 |  | 2 | 2 | 0 | 0 |
|  | Dismanters | 82 | 82 | 0 | 0 | 0 | 0 |  | 0 | 0 | 0 | 0 |  | 0 | 0 | 0 | 0 |
|  | Burners | 21 | 21 | 0 | 0 | 0 | 0 |  | 0 | 0 | 0 | 0 |  | 0 | 0 | 0 | 0 |
|  | Reference group | 41 | 40 | 0 | 0 | 0 | 1 |  | 1 | 0 | 0 | 1 |  | 1 | 0 | 1 | 0 |
|  | Combined | 217 | 214 | 0 | 1 | 0 | 2 |  | 3 | 2 | 0 | 1 |  | 3 | 2 | 1 | 0 |
| **Thigh, right** | Collectors | 73 | 68 | 1 | 0 | 3 | 1 |  | 6 | 2 | 1 | 3 |  | 5 | 1 | 4 | 0 |
|  | Dismanters | 82 | 80 | 0 | 0 | 2 | 0 |  | 2 | 0 | 2 | 0 |  | 2 | 1 | 0 | 1 |
|  | Burners | 21 | 21 | 0 | 0 | 0 | 0 |  | 0 | 0 | 0 | 0 |  | 3 | 1 | 2 | 0 |
|  | Reference group | 41 | 38 | 1 | 0 | 2 | 0 |  | 4 | 3 | 1 | 0 |  | 10 | 3 | 6 | 1 |
|  | Combined | 217 | 207 | 2 | 0 | 7 | 1 |  | 12 | 5 | 4 | 3 |  |  |  |  |  |
| **Knee, right** | Collectors | 73 | 35 | 8 | 7 | 22 | 1 |  | 40 | 20 | 9 | 11 |  | 38 | 13 | 10 | 15 |
|  | Dismanters | 82 | 59 | 9 | 7 | 6 | 1 |  | 24 | 12 | 9 | 3 |  | 23 | 14 | 4 | 5 |
|  | Burners | 21 | 16 | 2 | 1 | 1 | 1 |  | 5 | 1 | 3 | 1 |  | 5 | 0 | 5 | 0 |
|  | Reference group | 41 | 33 | 4 | 3 | 1 | 0 |  | 8 | 3 | 4 | 1 |  | 3 | 1 | 2 | 0 |
|  | Combined | 217 | 143 | 23 | 18 | 30 | 3 |  | 77 | 36 | 25 | 16 |  | 69 | 28 | 21 | 20 |
| **Lower leg & ankles, right** | Collectors | 73 | 39 | 5 | 5 | 23 | 1 |  | 34 | 14 | 11 | 9 |  | 32 | 9 | 8 | 15 |
|  | Dismanters | 82 | 71 | 4 | 2 | 5 | 0 |  | 10 | 5 | 4 | 1 |  | 11 | 5 | 2 | 4 |
|  | Burners | 21 | 19 | 0 | 2 | 0 | 0 |  | 2 | 0 | 1 | 1 |  | 2 | 1 | 1 | 0 |
|  | Reference group | 41 | 35 | 3 | 0 | 3 | 0 |  | 7 | 6 | 0 | 1 |  | 4 | 1 | 3 | 0 |
|  | Combined | 217 | 164 | 12 | 9 | 31 | 1 |  | 53 | 25 | 16 | 12 |  | 49 | 16 | 14 | 19 |
| **Lower leg, left** | Collectors | 73 | 39 | 5 | 5 | 23 | 1 |  | 34 | 14 | 11 | 9 |  | 32 | 9 | 8 | 15 |
|  | Dismanters | 82 | 71 | 4 | 2 | 5 | 0 |  | 10 | 5 | 4 | 1 |  | 10 | 5 | 2 | 3 |
|  | Burners | 21 | 19 | 0 | 2 | 0 | 0 |  | 2 | 0 | 1 | 1 |  | 2 | 1 | 1 | 0 |
|  | Reference group | 41 | 33 | 3 | 1 | 4 | 0 |  | 9 | 7 | 1 | 1 |  | 5 | 1 | 4 | 0 |
|  | Combined | 217 | 162 | 12 | 10 | 32 | 1 |  | 55 | 26 | 17 | 12 |  | 49 | 16 | 15 | 18 |
